# Predictors and long-term outcome of intracranial hemorrhage after thrombolytic therapy for acute ischemic stroke—A prospective single-center study

**DOI:** 10.3389/fneur.2023.1080046

**Published:** 2023-02-01

**Authors:** Klára Edit Fekete, Máté Héja, Sándor Márton, Judit Tóth, Aletta Harman, László Horváth, István Fekete

**Affiliations:** ^1^Department of Neurology, Faculty of Medicine, University of Debrecen, Debrecen, Hungary; ^2^Institute of Political Science and Sociology, Faculty of Arts, University of Debrecen, Debrecen, Hungary; ^3^Department of Radiology, Faculty of Medicine, University of Debrecen, Debrecen, Hungary; ^4^Department of Pharmaceutical Surveillance and Economics, Faculty of Pharmacy, University of Debrecen, Debrecen, Hungary

**Keywords:** thrombolysis, risk factors, intracranial hemorrhage, outcome, ischemic stroke

## Abstract

**Introduction:**

Acute ischemic stroke (AIS) is a potentially devastating disease with high disability and mortality. Recombinant tissue plasminogen activator (rt-PA) is an effective treatment with a 2–8% possible risk for symptomatic intracranial hemorrhage (sICH). Our aim was to investigate the risk factors and long-term clinical outcomes of ICH in patients after rt-PA treatment.

**Methods:**

Consecutive patients with AIS, thrombolysed at the Department of Neurology, University of Debrecen, between 1 January 2004 and 31 August 2016 were enrolled prospectively. Risk factors, stroke severity based on the National Institute of Health Stroke Scale (NIHSS), functional outcome using the modified Rankin scale, and mortality at 1 year were compared in patients with and without ICH following rt-PA treatment. We evaluated clinical characteristics and prognosis by hemorrhage type based on the Heidelberg Bleeding Classification. Descriptive statistics, the chi-square test, the Mann–Whitney *U*-test, ANOVA, the Kruskal–Wallis test, a survival analysis, and logistic regression were performed as appropriate.

**Results:**

Out of 1,252 patients with thrombolysis, ICH developed in 138 patients, with 37 (2.95%) being symptomatic. Mean ages in the ICH and non-ICH groups differed significantly (*p* = 0.041). On admission, the 24-h NIHSS after thrombolysis was higher in patients with ICH (*p* < 0.0001). Large vessel occlusion was more prevalent in patients with ICH (*p* = 0.0095). The ICH risk was lower after intravenous thrombolysis than intra-arterial or combined thrombolysis (*p* < 0.0001). Both at 3 months and 1 year, the outcome was worse in patients with ICH compared to patients without ICH group (*p* < 0.0001). Mortality and poor outcome were more prevalent in all hemorrhage types with a tendency for massive bleeding associated with unfavorable prognosis. At 3 months with the logistic regression model, the worse outcome was detected in patients with ICH after thrombolysis, at 1 year in patients with ICH after thrombolysis and smoking.

**Discussion:**

Older age, higher NIHSS, large vessel occlusion, and intra-arterial thrombolysis may correlate with ICH. The unfavorable outcome is more common in patients with ICH. Precise scoring of post-thrombolysis bleeding might be a useful tool in the evaluation of the patient's prognosis. Our findings may help to identify predictors and estimate the prognosis of ICH in patients with AIS treated with rt-PA.

## Introduction

Acute ischemic stroke (AIS) is a common and potentially devastating disease causing death in one-third of patients, leaving another third permanently disabled (1). Intravenous thrombolysis (IVT) with recombinant tissue plasminogen activator (rt-PA) is a standard treatment for AIS and has been proven as an effective and safe therapy within 3–4.5 h from the onset of stroke (2, 3). Local intra-arterial thrombolysis (IAT) was a possible therapeutic option in selected patients whose treatment could be started within 3–6 h after the onset of symptoms caused by the occlusion of the middle cerebral artery or within 12 h by occlusion of the basilar artery (4, 5). The significance of IAT changed over the years and nowadays. Intra-arterial thrombolysis can be used as a treatment of primary distal occlusions, as rescue after proximal occlusion thrombectomy, and/or as an adjunct therapy to primary mechanical thrombectomy (6). According to a recent summary, there is no clear consensus on best practices or criteria for the administration of IA rt-PA, although IAT is used in clinical practice (6). Nevertheless, trials concluded that IAT after thrombectomy is safe (7, 8). In mild strokes (NIHSSS ≤5) with LVO, the ICH rate in the case of IAT alone was better than after mechanical thrombectomy, emphasizing the importance of IAT (9).

Hemorrhagic complications, especially symptomatic intracranial hemorrhage (sICH), are the most feared and least treatable consequences of thrombolytic therapy, which may limit the use of rt-PA in patients with AIS. The risk of sICH varies from 2 to 8% depending on the definition used (based on NINDS, ECASS-II, III, ATLANTIS, and SITS-MOST studies) (10), while asymptomatic hemorrhagic transformation (HT) occurs in 18% (11). Several studies have demonstrated that HT after AIS is associated with poor functional outcomes and higher mortality rates (12). HT is a complex and multifactorial phenomenon that is most likely related to the disruption of the blood–brain barrier (BBB) and reperfusion injury of ischemic tissues (13). Several risk factors for sICH after thrombolysis have been identified. They include older age, greater stroke severity assessed by the National Institute of Health Stroke Scale (NIHSS), higher blood pressure on admission, history of diabetes mellitus, atrial fibrillation and baseline antithrombotic use, and the presence of acute ischemic changes in the computed tomography (CT) scan, all of which are proven poor prognostic factors (14). Knowledge of these predictors is important and may help clinicians to select the most suitable patients for treatment and improve the safety of thrombolysis.

The aim of our single-center prospective study was to evaluate the predictors and outcomes of ICH in patients having received thrombolytic therapy for AIS. In addition to the well-known risk factors, we analyzed the impact of large vessel occlusion (LVO) and the route of rt-PA administration for the incidence of ICH. We also evaluated the clinical characteristics and prognosis by hemorrhage type based on Heidelberg Bleeding Classification (15).

## Methods

### Subjects, patients

**Figure 1:**
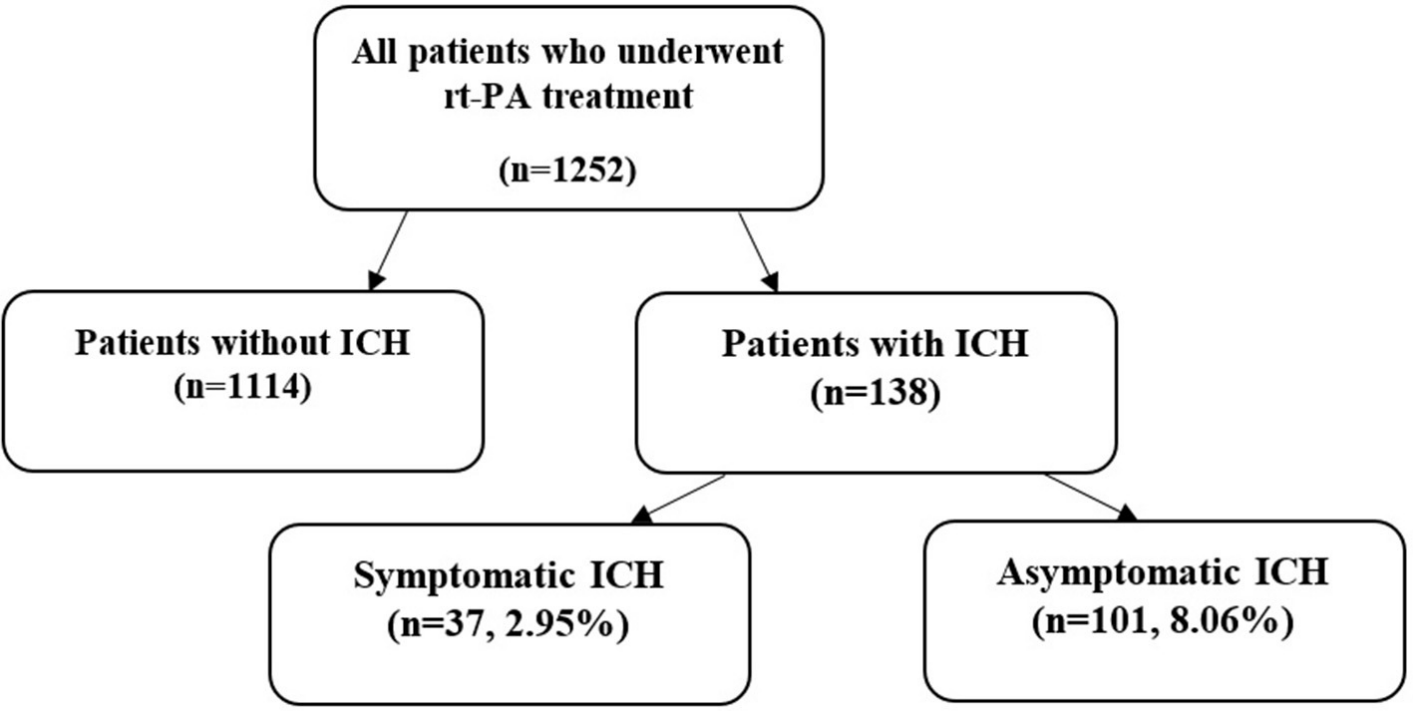
Flowchart of participants (rt-PA, recombinant tissue plasminogen activator; ICH, intracranial hemorrhage).

We performed a single-center prospective study. We analyzed 1,252 consecutive patients with AIS treated with rt-PA, of whom 1,124 had IVT, 61 patients underwent IAT, and 67 were given bridging therapy. Data were collected between 1 January 2004 and 31 August 2016. Our center receives patients within 90 km, in a catchment area of 600,000 inhabitants and 600–700 acute stroke hospitalizations per year. All of the patients were treated at the Neurological Intensive Care Unit, Department of Neurology, University of Debrecen, and we monitored the parameters recommended in the European Stroke Organization (ESO) guideline (5). Treatment for AIS with IV rt-PA started within 4.5 h after symptom onset was one of the inclusion criteria. The patients were categorized into two subgroups: patients with ICH and patients without ICH. In the latter group, 37 patients (2.95%) had symptomatic ICH, while 101 patients (8.06%) had asymptomatic ICH. Figure 1 shows a flowchart of participants.

### Database

The collected data included baseline characteristics, common stroke risk factors, prestroke anticoagulation, occurrence and location of large vessel occlusion, type of ICH, treatment modality, stroke severity, clinical outcome at 3 months, and mortality at 1 year.

We observed the prevalence of stroke risk factors as follows: hypertension, diabetes mellitus, previous or current smoking, history of stroke, atrial fibrillation, congestive heart failure, and alcohol abuse. Upon admission, we also checked every patient's blood glucose, cholesterol and triglyceride levels, and systolic and diastolic blood pressure. Blood pressure and laboratory parameters were expressed as mean ± standard deviation.

Stroke severity was assessed by the neurologist in the stroke unit, based on NIHSS on admission and 24 h later. NIHSS scores were presented as medians (1; 3 quartile).

The 3-month outcome was evaluated using the modified Rankin scale (mRS). A good clinical outcome was defined as a score of 0–2 (16). The assessment of mRS was performed during follow-up clinical visits by certified neurologists. At 1 year, we dichotomized patients into groups “dead” and “alive.”

We have chosen the risk factors of stroke (Table 1) and, on admission, the most important vital signs and stroke outcome for comparison.

**Table 1:** Baseline characteristics, risk factors, and clinical parameters of patients.

	**Patient groups**			
**Variable**	**Total** **(*****n** =* **1252)**	**ICH** **(*****n** =* **138)**	**no ICH** **(*****n** =* **1114)**	***P*** **-value**
Age (years), mean ± SD	67.7 ± 12.9	70 ± 10.3	67.5 ± 13.2	0.041
Gender, male, *n* (%)	702 (56)	84 (60)	618 (55.5)	NS
**Risk factors**				
Hypertension, *n* (%)	956 (76.3)	99 (71.7)	857 (77)	NS
Smoking, *n* (%)	318 (25.4)	38 (27.5)	280 (25.2)	NS
Diabetes mellitus, *n* (%)	248 (19.8)	24 (17.3)	224 (20.1)	NS
Alcohol abuse, *n* (%)	167 (13.3)	20 (14.5)	147 (13.2)	NS
History of stroke, *n* (%)	274 (21.9)	24 (17.4)	230 (20.7)	NS
Atrial fibrillation, *n* (%)	231 (18.4)	26 (18.8)	205 (18.4)	NS
Congestive heart failure, *n* (%)	165 (13.2)	19 (13.8)	146 (13.1)	NS
Pre-stroke anticoagulation, *n* (%)	122 (9.7)	17 (12.3)	105 (9.4)	NS
**Vital parameters on admission**				
Systolic blood pressure (mmHg), mean ± SD	156.7 ± 20.7	155 ± 25.54	158 ± 20.68	NS
Diastolic blood pressure (mmHg), mean ± SD	86.8 ± 13.27	86 ±17	113.5 ± 13.35	NS
Serum glucose level (mmol/l), mean ±SD	7.8 ± 1.8	7.25 ± 3.17	7.4± 2.7	NS
Cholesterol level (mmol/l), mean ± SD	5.17 ± 2.75	4.7 ± 0.49	4.9 ± 1.04	0.053
Triglyceride level (mmol/l), mean ± SD	1.27 ± 0.91	1.15 ± 0.34	1.56 ± 0.91	NS
NIHSS score on admission, median (1; 3 quartile)	10 (6; 16)	14 (10; 18)	10 (5; 15)	<0.0001

### Imaging

All patients underwent non-contrast CT on admission. Arterial occlusion (trunk or at least one branch of any large artery) was identified by CT angiography. Where available (93.7%), the Alberta Stroke Programme Early CT Score (ASPECTS) was used to assess early ischemic signs on admission (17). The CT was repeated 24 h after treatment in the case of clinical relapse to evaluate hemorrhagic changes. Any hemorrhage detected intracranially with imaging within 24 h after treatment was defined as post-thrombolysis ICH (18). We rated all follow-up CT scans based on the anatomical description of ICH according to the Heidelberg Bleeding Classification, where hemorrhagic infarction (HI) and parenchymatous hematoma (PH) were used as basic: *HI-1* refers to the hemorrhagic transformation of infarcted tissue as scattered small petechiae without mass effect (class 1a); *HI-2* is more confluent petechiae within the infarcted area but without space-occupying lesion (class 1b); *PH-1* is defined as a hematoma not exceeding 30% of the infarcted area but with some mild space-occupying lesion (class 1c); *PH-2* represents hematoma occupying 30% or more of the infarcted tissue, with an obvious mass lesion (class 2). Other bleeding types are classified as ICH outside the infarcted tissue (class 3a), intraventricular (class 3b), subarachnoid (class 3c), or subdural (class 3d) hemorrhage (15). In **Table 3**, we have chosen the variables according to our preliminary data that might have had an impact on the form of bleeding and the outcome in different groups was also of interest. The following parameters were compared in the categories of the Heidelberg Bleeding Classification score: age, ASPECTS on admission and at 24 h, NIHSS score on admission and at 24 h, serum glucose level, mRS at 3 months, and sICH.

Patients with ICH were categorized into sICH and asymptomatic ICH groups. We used three definitions for sICH: SITS, ECASS, and RCT NINDS criteria (2, 19, 20), while asymptomatic ICH was defined as the presence of any hemorrhage without neurological worsening (21).

### Treatment

Intravenous thrombolysis was performed in accordance with the ESO guidelines (5, 22). In the case of IVT, the total amount of rt-PA was 0.9 mg/kg of body weight (maximum 90 mg), with 10% of the dose given as a bolus followed by an infusion over 60 min using a syringe pump. Continuous monitoring of neurological status, pulse, blood pressure, body temperature, and oxygen saturation was performed according to guideline recommendations. Patients diagnosed with large vessel occlusion were started on intravenous treatment which was followed by intra-arterial administration (“bridging” therapy). IAT was used alone in patients who were not candidates for IV rt-PA. A microcatheter (Progreat TERUMO) was used for endovascular intervention. The microcatheters used were compatible with “0.017 and 0.021” guide wires. We navigated it at the site of the occlusions until the occlusion, and repeated doses of 5 mg rt-PA were given by electric syringe (1 mg/min) until the artery opened up or the maximum weight-adjusted dose was reached. After every 5 mg of rt-PA contrast material was given, and if the vessel did not open, we continued the procedure. The study and the intra-arterial use of rt-PA were approved by the Local Research Ethics Committee of the University of Debrecen (23). In 11 cases—among which only one patient had ICH—another therapeutic approach, mechanical thrombectomy, was used, when it was already available. This was negligible compared to other interventions. The type of treatment was chosen according to the actual guidelines and individually decided which treatment modality was used by the treating physician who consulted with the neuroradiologists.

### Statistical analysis

Statistical analysis was carried out using the SPSS for Windows 19.0 program suite (SPSS Inc. Chicago, USA). Descriptive statistics were performed. Correlations between categorical variables were identified using Pearson's chi-squared test, and correlations between continuous variables were determined using the Mann–Whitney *U*-test. To compare each hemorrhagic transformation group, we used the Kruskal–Wallis test for non-parametric variables and the one-way ANOVA test for metric variables. The binary logistic regression analysis was used to assess outcomes at 3 months and at 1 year. Logistic regression models were used to identify the independent predictors of 3-month disability and 1-year case fatality. The analysis was performed with the multivariate general linear model (GLM). In the models, disability at 3 months (mRS >2) and case fatality at 1 year were the dependent variables, and those factors that were found to be associated with the outcome by univariate analyses were entered as confounding variables. The variables were excluded from the analysis one by one, and the variable with *p* > 0.05 and closest to 1.0 dropped out, until all features left in the model had *p* < 0.05. Survival analyses were done (Kaplan–Meier curve and logrank).

All tests were performed at a *p*-value of < 0.05 significance level.

## Results

### Baseline characteristics

The baseline characteristics, risk factors, and clinical parameters of the patients are summarized in Table 1. A total of 1,252 patients, 702 male patients (56%) and 550 (44%) female patients, with AIS received rt-PA treatment (aged 17–99 years; mean age, 67.7 ± 12.9). ICH was detected in 138 patients (11%); sICH occurred in 37 patients (2.95%), while asymptomatic ICH affected 101 patients (8.06%). Out of these 138 patients, 94 had ischemia-related HT, six patients had intracerebral hemorrhage outside the infarcted tissue, 26 had subarachnoid hemorrhage, 11 had intraventricular hemorrhage, and only one patient had subdural hematoma. The patients with ICH were significantly older than those without ICH (70 ± 10.3 vs. 67.5 ± 13.2, *p* = 0.041).

None of the analyzed stroke risk factors showed significant differences between the two groups. Compared with the non-ICH group, patients with ICH presented with lower serum cholesterol levels on admission (4.9 ± 1.04 vs. 4.7 ± 0.49 mmol/l), but the difference was not significant (*p* = 0.053). Baseline stroke severity was significantly higher (*p* < 0.0001) in patients with ICH compared to patients without ICH [median NIHSS scores on admission were 14 (10, 18) and 10 (5, 15), respectively].

### Imaging

**Figure 2:**
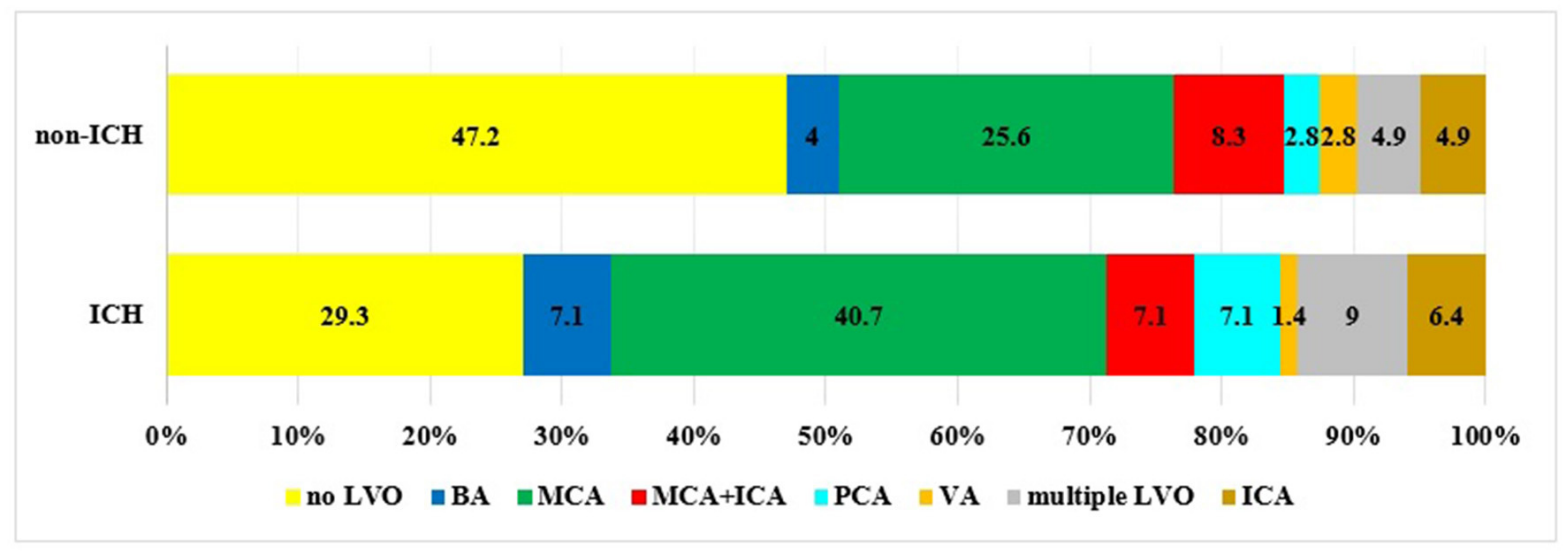
Incidence of LVO in patients with ICH compared to patients without ICH (BA, basilar artery; MCA, middle cerebral artery; ICA, internal carotid artery; PCA, posterior cerebral artery; VA, vertebral artery).

Figure 2 demonstrates the occurrence of arterial occlusion in patients with ICH and without ICH. For the entire patient population, LVO was detected in 688 patients (54.9%). The incidence of LVO was significantly higher in the ICH group compared to the non-ICH group (70.7% vs. 52.8%, *p* = 0.0095). ICH was more/most likely to develop as a result of an occlusion in the middle cerebral artery (40.7% vs. 25.6%, *p* = 0.0062), the basilar artery (7.1% vs. 4%, *p* = 0.097), and the posterior cerebral artery (7.1% vs. 2.8%, *p* = 0.15) or in the case of multiple arterial occlusions (9% vs. 4.9%, *p* = 0.27).

### Treatment modality

Regarding the route of rt-PA administration, IVT occurred in 1,124 patients, 61 patients received IAT, while “bridging” therapy was done in 67 patients. In the ICH group, the distribution of treatment modalities was as follows: IVT and IAT were given to 100 patients (72.5%) and 24 patients (17.4%), respectively, while 14 patients (10.1%) received combined therapy. The relevant percentages in the non-ICH group were 89.7, 4.9, and 5.4%, respectively. The risk of ICH was 9.1% in intravenous thrombolysis, significantly lower than in intra-arterial (39.3%) or combined thrombolysis (20.9%) including mechanical thrombectomy. These data show that the intra-arterial use of rt-PA is associated with a significantly higher rate of ICH (*p* < 0.0001).

### Outcome

**Figure 3:**
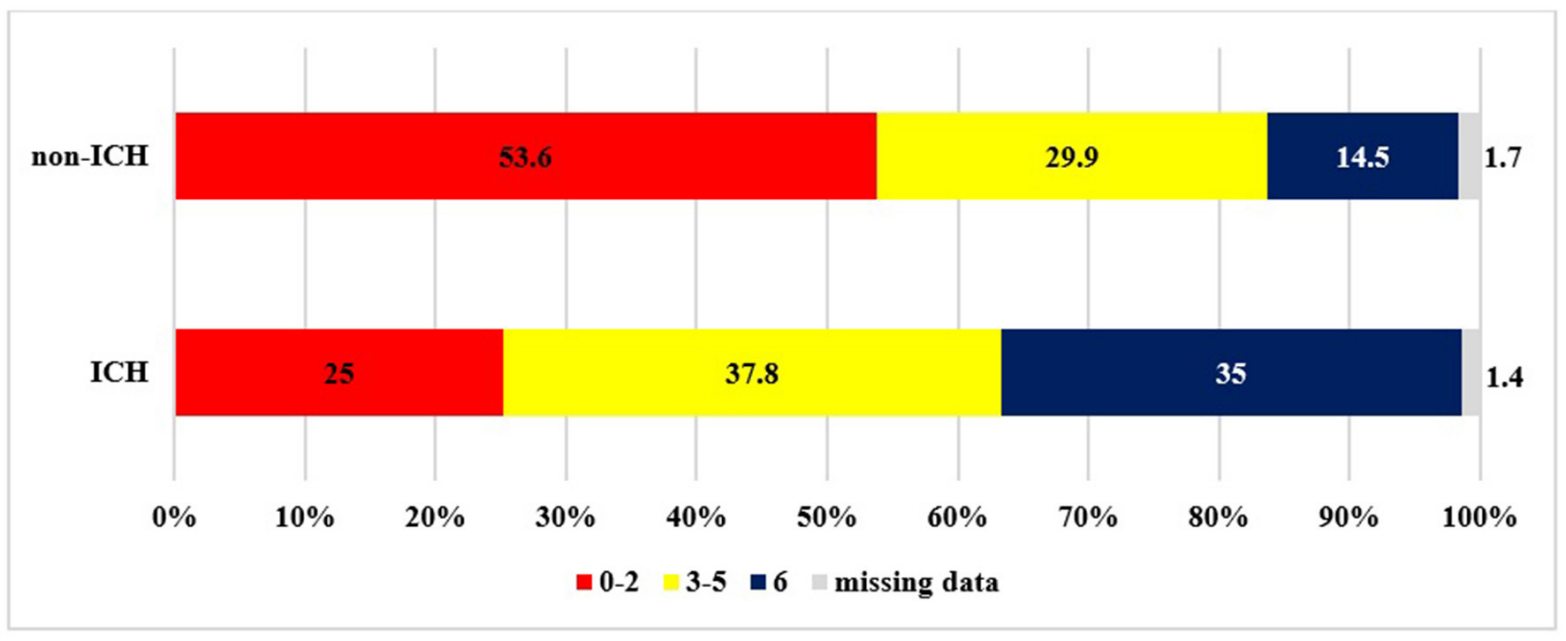
Outcome at 3 months based on mRS (ICH: intracranial hemorrhage).

**Figure 4:**
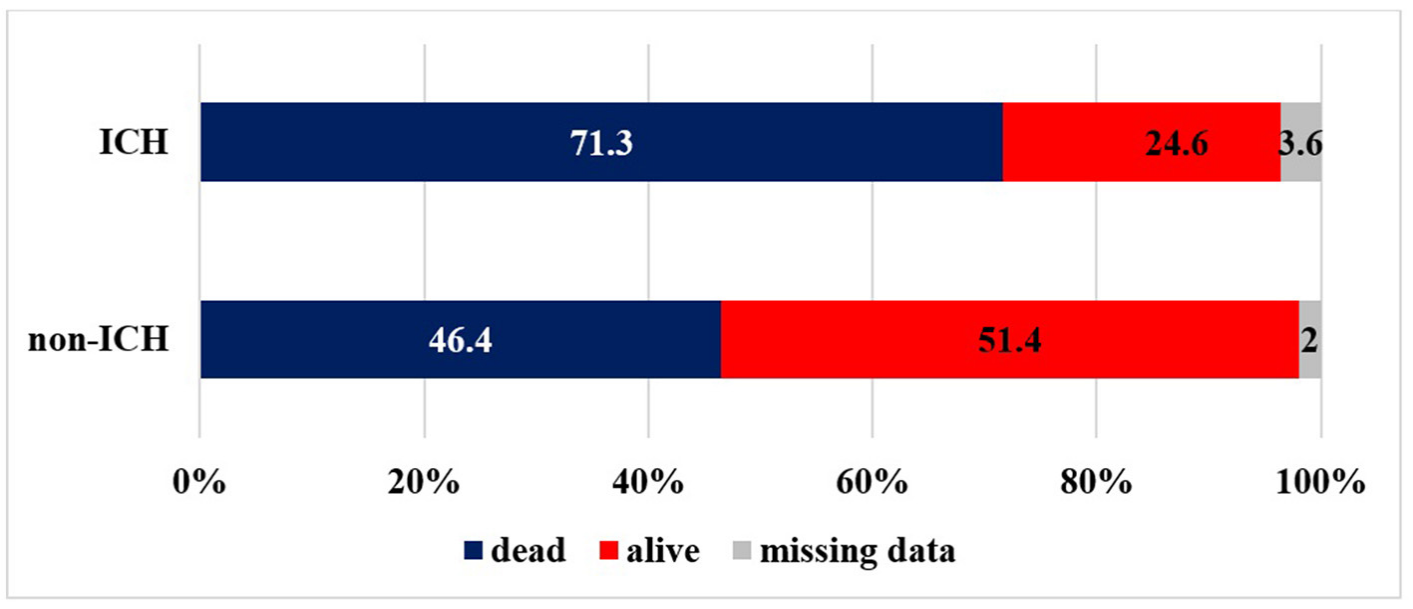
Mortality at year (ICH, intracranial hemorrhage).

Table 2 and Figures 3, 4 summarize the data concerning clinical outcomes. At 24 h, the patients in the ICH group had higher NIHSS scores than patients without ICH [median NIHSS scores 15 (9, 20) and 7 (3, 14)], the difference being statistically significant (*p* < 0.0001). At 3 months, only 26 % of the patients with ICH had favorable outcomes (mRS: 0–2), which was significantly worse (*p* < 0.0001) compared to the non-ICH group (53.6%). More than one-third of the patients with ICH (36.9%) had moderate or severe residual symptoms (mRS: 3–5) and, unfortunately, 35.5% of patients were dead at 3 months. In the non-ICH group, however, those rates were 29.8 and 14.5%, respectively. The differences also were significant (*p* < 0.0001). At 1 year, 52.2% of the patients with ICH had passed away, while 23.6% of the patients without ICH had passed away (*p* < 0.0001).

**Table 2:** Clinical outcome of patients with and without post-thrombolysis intracranial hemorrhage.

	**ICH (*n =* 138)**	**non-ICH (*n =* 1114)**	***P*-Value**
NIHSS score at 24 h, median (1; 3 quartile)	15 (9; 20)	7 (3; 14)	<0.0001
mRS score at 3 months			<0.0001
Favorable outcome (mRS: 0-2), *n* (%)	36 (26)	598 (53.6)	
Moderate/severe disability (mRS: 3-5), *n* (%)	51 (36.9)	333 (29.8)	
Death (mRS: 6), *n* (%)	49 (35.5)	162 (14.5)	
Mortality at one year	72 (52.2)	263 (23.6)	<0.0001

**Figure 5:**
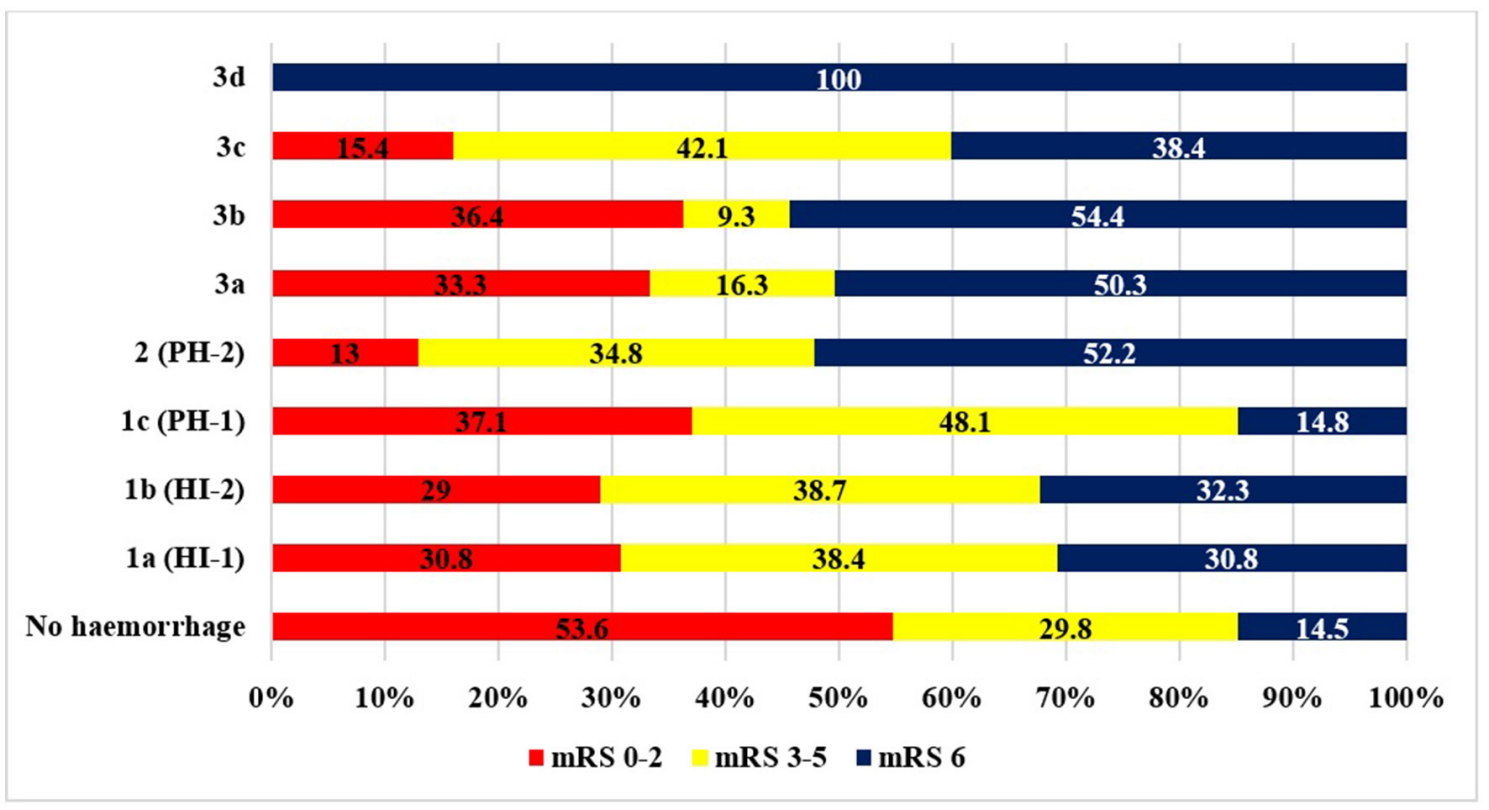
Outcome at 3 months on the mRS by hemorrhage type. Abbreviations are defined in the text.

Table 3 and Figure 5 show the baseline characteristics, the functional outcome at 90 days, and the rate of sICH by the extent of thrombolysis-related hemorrhagic transformation. HI-2 was more frequent than HI-1, PH-1, and PH-2. Patients who experienced HT were of advanced age, especially in the PH-2 and class 3a groups (*p* = 0.04). Elevated serum glucose levels were more likely in patients with HT, especially in the HI-1 group and in patients with intraventricular hemorrhage (*p* = 0.019). The means of ASPECTS on admission did not differ significantly in the groups, but with follow-up CT scans at 24 h, significantly lower ASPECT scores were identified in all HT groups. PH-1 and PH-2 were more frequently associated with higher baseline NIHSS scores when compared with no HT (*p* = 0.0004). At 24 h, the median NIHSS was significantly higher in all HT groups. Mortality and poor outcome were more prevalent in all hemorrhage types with a tendency for heavy/massive bleeding associated with unfavorable prognosis. The rates of sICH did not differ significantly in the HT groups.

**Table 3:** Baseline characteristics, functional outcome at 3 months, and rate of sICH in patient groups based on Heidelberg bleeding score.

	**Patient groups based on Heidelberg bleeding score**									
**Variable**	**No HT**	**1a** **(HI-1)**	**1b** **(HI-2)**	**1c** **(PH-1)**	**2** **(PH-2)**	**3a**	**3b**	**3c**	**3d**	***P*** **-value**
**N**	1114	13	31	27	23	6	11	26	1	**<0.001**
**Age (years), mean** **±SD**	67.5 ± 13.2	70.2 ± 10.3	70.3 ± 11.6	67 ± 8.8	72.8 ± 8.7	72 ± 9	70 ± 10.3	69.8 ± 13.2	77	**0.040**
**ASPECTS on admission, mean**	9.1	9.5	8.1	8.6	9.4	10	8.13	9.13	10	0.965
**ASPECTS at 24h, mean**	7.1	5.3	5.9	5.0	4.7	4.5	5.2	4.78	3	**0.007**
**NIHSS score on admission, median** **(1; 3 quartile)**	10 (5; 15)	10.5 (6; 15)	10 (7.75; 16)	14 (12; 18.5)	16 (12.5; 18.5)	10 (10; 13.75)	16 (10.5; 17.5)	14 (11; 16.75)	14	**0.0004**
**NIHSS score at 24h, median** **(1; 3 quartile)**	7 (3; 14)	14 (8.75; 16.5)	11 (6.75; 17)	15 (5; 19)	19 (15; 22.5)	15.5 (12; 18.25)	17 (13.5; 20)	16.5 (11.25; 21)	45	**<0.0001**
**Serum glucose level (mmol/l), mean** **±SD**	7.4 ± 2.7	9.47 ± 5.3	8.3 ± 3	8.1 ± 3.6	8.2 ± 2.8	8.3 ± 5	9.3 ± 3.7	7.5 ± 2.8	5.5	**0.019**
**mRS score at 3 months**, ***n*** **(%)**										
**Favorable outcome** **(mRS: 0-2)**	598 (53.6)	4 (30.8)	9 (29)	10 (37.1)	3 (13)	2 (33.3)	4 (36.4)	4 (15.4)	0 (0)	**<0.00001**
**Moderate/severe disability** **(mRS: 3-5)**	333 (29.8)	5 (38.4)	12 (38.7)	13 (48.1)	8 (34.8)	1 (16.3)	1 (9.3)	11 (42.1)	0 (0)	**0.003**
**Death (mRS: 6)**	162 (14.5)	4 (30.8)	10 (32.3)	4 (14.8)	12 (52.2)	3 (50.3)	6 (54.4)	10 (38.4)	1 (100)	**<0.00001**
sICH, *n* (%)	-	2 (15.4)	9 (28.1)	6 (22.2)	4 (17.4)	3 (50)	4 (36.4)	8 (30.8)	1 (100)	0.17

Abbreviations are defined in the text. The bold values indicate the values of *p* < 0.05.

**Figure 6:**
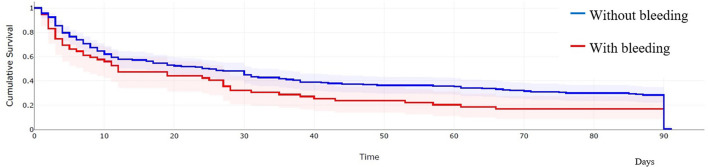
Kaplan–Meier curve of the death given in days in patients with and without bleeding after thrombolysis.

Survival analyses were conducted, but there were limitations to their interpretation (Figure 6). The 3-month survival rate in the group with intracerebral hemorrhage was significantly different from that of the group with no hemorrhage. Between 3 months and 1 year, the difference was not significant. The sample size was too small for a logrank test, so we could not perform a correct evaluation. The other factor limiting the interpretation was that the exact dates of deaths between 3 months and 1 year had not always been correctly recorded. The records only informed about whether a patient died within 3 months or within 1 year.

With the logistic regression model (Table 4) at 3 months, a significant difference was detected in patients with diabetes mellitus, previous atrial fibrillation, previous cardiac failure, and intracerebral bleeding after thrombolysis. From them, intracerebral bleeding after thrombolysis was the variable that had a major negative impact on the outcome at 3 months and at 1 year not only ICH but smoking also. Interestingly, diabetes mellitus, previous atrial fibrillation, and previous cardiac failure seem to have a positive effect in the logistic regression model, but this might be due to the relatively small sample size in some subgroups. Another possible explanation might be that these groups were treated previously, so their conditions were known and treated, and secondary prevention has been applied.

**Table 4:** Factors that have a significant effect on the outcome (poor outcome means mRS > 2 points) at 3 months and at 1 year with the logistic regression model.

	**3 months**			**1 year**		
**Factors**	***p***	**EXP(B)**	**95% Confidence interval CI**	***p***	**EXP(B)**	**95% Confidence interval CI**
Diabetes mellitus	**0.019**	**0.692**	**(0.508; 0.942)**	0.141	0.781	(0.563; 1.085)
Smoking	0.075	1.078	(0.822; 1.414)	**<0.0001**	**1.703**	(1.239; 2.342)
Previous atrial fibrillation	**0.005**	**0.623**	**(0.448; 0.866)**	0.355	0.528	(0.379; 0.737)
Previous cardiac failure	**0.004**	**0.570**	**(0.387; 0.838)**	**0.001**	**0.513**	(0.350; 0.753)
Previous stroke	**0.008**	**0.661**	**(0.485; 0.899)**	0.355	0.856	(0.615; 1.190)
ICH after thrombolysis	**<0.0001**	**3.454**	**(2.262; 5.267)**	**<0.0001**	**3.168**	(2.144; 4.682)

The bold values indicate the values of *p* < 0.05.

## Discussion

Currently, rt-PA is the only approved and validated treatment for pharmacological revascularization in AIS. The treatment option for rt-PA has been selected according to the current guidelines for IV treatment; at the time of our study, IAT could be given according to the ESO 2008 guideline (5), but today IA is given after mechanical thrombectomy in the frame of studies (6–8) or alone in mild acute ischemic strokes (9). The majority of patients undergoing thrombolysis have a good prognosis compared with patients not receiving thrombolysis (3). However, the use of thrombolytic therapy is associated with an increased risk of ICH, which can reduce the chances of favorable outcomes. In this prospective single-center study, we analyzed the data of a total of 1,252 patients with AIS having undergone thrombolysis. We estimated the incidence, predictors, and outcome of ICH after treatment.

The rate of sICH in our study was 2.95%, while asymptomatic ICH occurred in 8.06%, which, in line with previous international studies, shows that it is one of the most common and serious complications of thrombolysis (20, 24, 25). Many of the underlying mechanisms for HT have not been completely evaluated yet, but studies have suggested that reperfusion injury, oxidative stress, leukocyte infiltration, vascular activation, and dysregulated extracellular proteolysis are the principal triggers for the disruption of BBB which leads to blood extravasation (26). It is also suggested that the use of rt-PA may exacerbate BBB disruption by activating matrix metalloproteinases and altering endothelial function (27). A number of clinical, radiological, and laboratory variables have been shown to be associated with an increased risk of sICH following thrombolysis. In our study, the results of statistical analysis indicated that older age, higher NIHSS, LVO, and intra-arterial administration of rt-PA were risk factors for ICH after thrombolytic therapy.

Age is the most remarkable non-modifiable risk factor for stroke and a major predictor of clinical outcome (28). The literature on the risk of sICH after thrombolysis in the elderly is divisive. Previously, several studies have shown that advanced age is an independent risk factor for sICH (19, 29, 30) and was considered a relative contraindication for 3–4.5 h IVT by many guidelines. However, many other articles have reported that the incidence of sICH does not differ significantly between younger and older patients, and this age group still seems to benefit from treatment, as was mentioned in our previous publication (31–34).

In the present study, the patients with ICH were significantly older than those without ICH. At the same time, in our previous study, when patients with thrombolysis were dichotomized into those aged ≥ 80 years and those aged <80 years, statistically speaking, the risk of sICH was not significantly greater in the older group (34). These data suggest that patients over 80 and suffering from an acute stroke should not be excluded from treatment with rt-PA based on their risk for sICH.

Although a history of hypertension, being the most significant pre-existing risk factor, was present in 76.3% of the patients in the overall population, we could not find any significant correlation in either the presence of hypertension or the mean blood pressure values on admission between the ICH and non-ICH groups. Other comorbidities, including diabetes mellitus, previous stroke, smoking, atrial fibrillation, prestroke anticoagulation, congestive heart failure, and alcohol consumption, were not correlated with ICH in the present study. However, we found that elevated baseline blood glucose was more likely in the HI and PH subgroups, which was consistent with previous findings where it had been shown that serum glucose was a predictor of ICH in patients treated with rt-PA (35). Some reports suggest that lower serum total cholesterol and triglyceride levels are associated with an increased risk for ICH (36, 37). We also found a similar trend, although no statistical significance was reached (*p* = 0.053). A possible explanation for this relationship is that cholesterol plays an important role in the integrity of small cerebral vessels and the neurovascular unit (23).

It is an important finding of our study that the NIHSS scores at presentation and at 24 h are significantly higher in patients with ICH. NIHSS measures stroke severity, which is primarily associated with the size of cerebral infarction. The higher the NIHSS score values are, the more severe the strokes and the larger the infarcts may become. Most of the previous studies including NINDS and ECASS trials have also shown that the severity of a baseline stroke is one of the most important predictors of ICH after thrombolysis (38). In our study, subgroup analyses across the HT groups showed that PH-1 and PH-2 were associated with higher baseline NIHSS scores than the HI groups. It has been suggested that the underlying mechanism of HI and PH differs from each other. Previous studies revealed that HI frequently occurred without the use of rt-PA as a natural phenomenon in the course of ischemic stroke, while severe PH was mainly associated with treatment using alteplase (39, 40). It can be an explanation for our finding that severe ischemic stroke with higher baseline NIHSS is suggestive of larger areas of infarcted tissue, including injured blood vessels, which are more likely to bleed after thrombolytic treatment. However, in our study, the median NIHSS scores at 24 h were significantly higher in all HT groups compared to no HT. These data suggest that post-thrombolysis hemorrhage is associated with early deterioration of neurological symptoms regardless of the extent of bleeding.

In the present study, the incidence of LVO was found to be 54.9%, slightly higher than the rate (24–46%) reported in the literature (41). Our results showed that LVOs, especially occlusions in the middle cerebral artery (MCA), were significantly more prevalent in patients with ICH, as also found in previous studies (42, 43). The MCA is the largest intracranial artery and by far the most commonly affected vessel in AIS. The occlusion of this artery may lead to massive cerebral infarction which is one of the most dangerous factors of HT. The development of extensive brain edema and enhanced permeability of vascular walls caused by prolonged ischemia and hypoxia may be the possible explanation for the higher probability of HT (24). The use of rt-PA can aggravate BBB disruption and may further increase the risk of HT (27). Therefore, the presence of an LVO shows a positive correlation with the incidence of ICH after thrombolysis.

Regarding the route of administering rt-PA, we found that the hemorrhage rate in patients treated with IVT was comparable to the rates reported in large thrombolysis trials (2, 20, 25). Another conclusion of our study is that intra-arterial use of rt-PA is associated with a significantly higher rate of ICH, which is also consistent with previous findings (24, 43). Large multicenter studies (PROACT II, MELT, and IMS II) have proven the efficacy of performing intra-arterial thrombolysis in LVO, although the indications and dosage of IAT remained less well-standardized (44). The rate of ICH in the aforementioned studies has ranged from 10 to 15%, which is in agreement with our results (11%). In the CHOICE trial, IAT was given after mechanical thrombectomy—this is different from our scenario because our patients with IAT did not undergo mechanical thrombectomy—in 61 patients (vs. 52 placeboes), sICH was 0% compared with placebo, but overall cerebral hemorrhage was 19% (6). A possible reason for the increased risk of ICH is the relatively high concentration of the thrombolytic agent at the site of application, which triggers a stronger activation of metalloproteinases and greater damage to the BBB (45); in addition, this is the larger infarct side that might occur in case of LVO. Nowadays, the first choice of LVO treatment is thrombectomy, but rt-PA treatment should be started, if needed before it, emphasizing the impact of our study in everyday use.

Finally, as far as the long-term outcome is considered, the functional status at 3 months has turned out to be significantly worse in the ICH group. This result is consistent with most previous studies which showed lower rates of favorable and independent outcomes in patients with ICH at day 90 (25, 29, 31, 46). Similarly, the 1-year mortality rate is also significantly higher in patients with ICH. Based on these studies, the fatality rates are between 50 and 80%, which is consistent with our result (50.7%). However, two factors need to be mentioned regarding the effect of ICH on the outcome. First, it is still unclear how the extent of HT influences the long-term functional outcome. An ECASS-II study reported that none of the radiological subtypes of hemorrhage have the same effect on the outcome (19). There is no doubt that massive HT is likely to be associated with clinical worsening/relapse, while HI might be a clinically irrelevant phenomenon of ischemic damage and reperfusion (39). Our results showed that both HI and PH have a negative impact on patient outcomes, which is consistent with some previous studies (46, 47) suggesting that HI grades of HT may not be benign. Second, there is an overlap between risk factors for thrombolysis-associated ICH and risk factors for poor outcomes following thrombolytic therapy in the absence of ICH. Thus, the observed poor outcomes are more probably related to the combination of the ICH and the underlying ischemic event itself (14).

The logistic regression model shows the importance of regular screening of the population suffering from diabetes mellitus, atrial fibrillation, cardiac failure, stroke, and quitting smoking. Intracerebral bleeding was a strong predictor of worse outcomes even in these models at 3 months and at 1 year as well. Our aim was to find the predictors of ICH after rt-PA treatment, including the treatment modality. Our findings show that not only LVO but IA treatment alone is also a risk factor. Many factors, such as technique and equipment, may be responsible for this, but our study was not designed to find this. Our study was a real-life scenario study with consecutive and not randomized patients, so the findings may be different from randomized clinical trials.

Our study has limitations. First, the size of the sample is relatively small for survival analysis. In the Heidelberg Bleeding Classification, some groups had few patients. Nevertheless, the most important and relevant risk factors have been identified in a real-world scenario. Second, we could not form a parallel non-treated group for obvious ethical reasons. If we had managed to, it could have ruled out the bias that, without rt-PA treatment, ICH might also occur in AIS. Despite the aforementioned, our study has highlighted that ICH after treatment may be a prognostic factor for poor outcomes.

## Conclusion

In conclusion, we found that patients of older age, having higher NIHSS, suffering from an occlusion affecting a large intracranial artery, and treated with intra-arterial rt-PA were at an increased risk for ICH after thrombolysis. Those patients seemed to have worse long-term functional outcomes and higher mortality rates than patients without ICH, so ICH after thrombolysis was a strong predictor of worse outcomes in univariate and multivariate analyses as well. We found that precise scoring of post-thrombolysis bleeding might be a useful tool in the evaluation of the patient's prognosis. To our knowledge, this was the first study with a real-life scenario in our region on this patient population.

## Data availability statement

The raw data supporting the conclusions of this article will be made available by the authors, without undue reservation.

## Ethics statement

The studies involving human participants were reviewed and approved by the Local Research Ethics Committee of the University of Debrecen. Written informed consent for participation was not required for this study in accordance with the national legislation and the institutional requirements.

## Author contributions

KF, IF, and LH led the initiative and revised the drafted document. KF, LH, and MH selected the abstract, extracted data, and drafted the manuscript. KF, AH, and LH were involved in the creation of the database. JT was involved in the evaluation and conceptualization of the radiological part. SM, LH, KF, and MH were involved in the investigation, data curation, data analysis, and writing the original draft. IF and KF were involved in supervision. All authors were involved in the conceptualization, methodology, review, editing and approved the final version.
